# Application of the child community health inclusion index for measuring health inclusion of children with disabilities in the community: a feasibility study

**DOI:** 10.1186/s12887-023-03884-8

**Published:** 2023-02-20

**Authors:** Paul Yejong Yoo, Annette Majnemer, Robert Wilton, Sara Ahmed, Keiko Shikako

**Affiliations:** 1 School of Physical and Occupational Therapy, Faculty of Medicine and Health Sciences, McGill University, 3500 Blv Decarie, Room 439, Montreal, QC H4A3J5 Canada; 2 Research Institute of the McGill University Health Centre, Montreal, QC H4A 3J1 Canada; 3 School of Geography & Earth Sciences, McMaster University, Hamilton, ON L8S 4L8 Canada; 4 Faculty of Social Sciences, McMaster University, Hamilton, ON L8S 4L8 Canada

**Keywords:** Community inclusion, Childhood disability, Measurement, Feasibility, Participation

## Abstract

**Background:**

Participation in the community is a fundamental human right for children with disabilities and is a key component of their health and development. Inclusive communities can enable children with disabilities to participate fully and effectively. The Child Community Health Inclusion Index (CHILD-CHII) is a comprehensive assessment tool developed to examine the extent to which community environments foster healthy, active living for children with disabilities.

**Objectives:**

To assess the feasibility of applying the CHILD-CHII measurement tool across different community settings.

**Methods:**

Participants recruited through maximal representation, and purposeful sampling from four community sectors (Health, Education, Public Spaces, Community Organizations) applied the tool on their affiliated community facility. Feasibility was examined by assessing length, difficulty, clarity, and value for measuring inclusion; each rated on a 5-point Likert scale. Participants provided comments for each indicator through the questionnaire and a follow-up interview.

**Results:**

Of the 12 participants, 92% indicated that the tool was ‘long’ or ‘much too long’; 66% indicated that the tool was clear; 58% indicated that the tool was ‘valuable’ or ‘very valuable’. No clear consensus was obtained for the level of difficulty. Participants provided comments for each indicator.

**Conclusion:**

Although the length of the tool was regarded as long, it was seen to be comprehensive and valuable for stakeholders in addressing the inclusion of children with disabilities in the community. The perceived value and the evaluators’ knowledge, familiarity, and access to information can facilitate use of the CHILD-CHII. Further refinement and psychometric testing will be conducted.

**Supplementary Information:**

The online version contains supplementary material available at 10.1186/s12887-023-03884-8.

## Introduction

The United Nations (UN) Convention on the Rights of the Child (CRC) and the Convention on the Rights of Persons with Disabilities (CRPD) call for the full and effective participation and inclusion of children with disabilities in society [1, 2]. These conventions outline the need for equal access to built environments, public transportation, information and communications technology, and other programs and services for children with disabilities to participate fully and effectively [1, 2]. Participation in community life has been found to be important for the health and development of competency, identity, and self-sufficiency of children with disabilities [3].

The Community Health Inclusion Index (CHII) is a comprehensive measurement tool that was developed to be used by community stakeholders to examine the scope and depth of factors that foster healthy, active living among people with disabilities [4]. This measure evaluates potential barriers and facilitators in the community that may influence the participation of individuals with disabilities. Previous studies underscored the need for a tailored tool that examines the inclusion of children with disabilities in the community spaces they occupy [5, 6]. This warranted the adaptation of the CHII to the pediatric population in the Canadian context.

Through an extensive literature review, expert panel consultation, and a systematic iterative process, the content of the CHILD-CHII was developed [5]. The content was then validated through a modified e-Delphi technique with input from diverse stakeholders [6]. These procedures further highlighted the need for and importance of a tool specifically tailored for children that consider the diverse and evolving need of children with various types of disabilities [5]. The tool itself assesses key environmental and community aspects that address social determinants of health promoted by the World Health Organization and Public Health Agency of Canada [6]. The tool can be used in clinical practice, community and public policy development, to support communities in identifying areas for improvement regarding the inclusion of children with disabilities within their facilities and facilitate their participation [5, 6].

Following the steps in content development and validation of the CHILD-CHII measurement tool, a feasibility study was the appropriate subsequent step [7]. A feasibility study is beneficial as it can suggest the potential usefulness of the tool and the feasibility of its use for further development [8]. Therefore, the objective of this study was to examine the feasibility of applying the CHILD-CHII measurement tool on community facilities by parents, clinicians, government, and community workers.

## Methods

### Study design

This feasibility study was conducted following the guidelines for reporting non-randomised pilot and feasibility studies for development of patient-reported outcome measures [7–9]. This study was approved by the McGill University Institutional Ethics Review Board and the Center for Interdisciplinary Research in Rehabilitation of the Greater Montreal Ethics Review Board.

### Participants

**Fig. 1:**
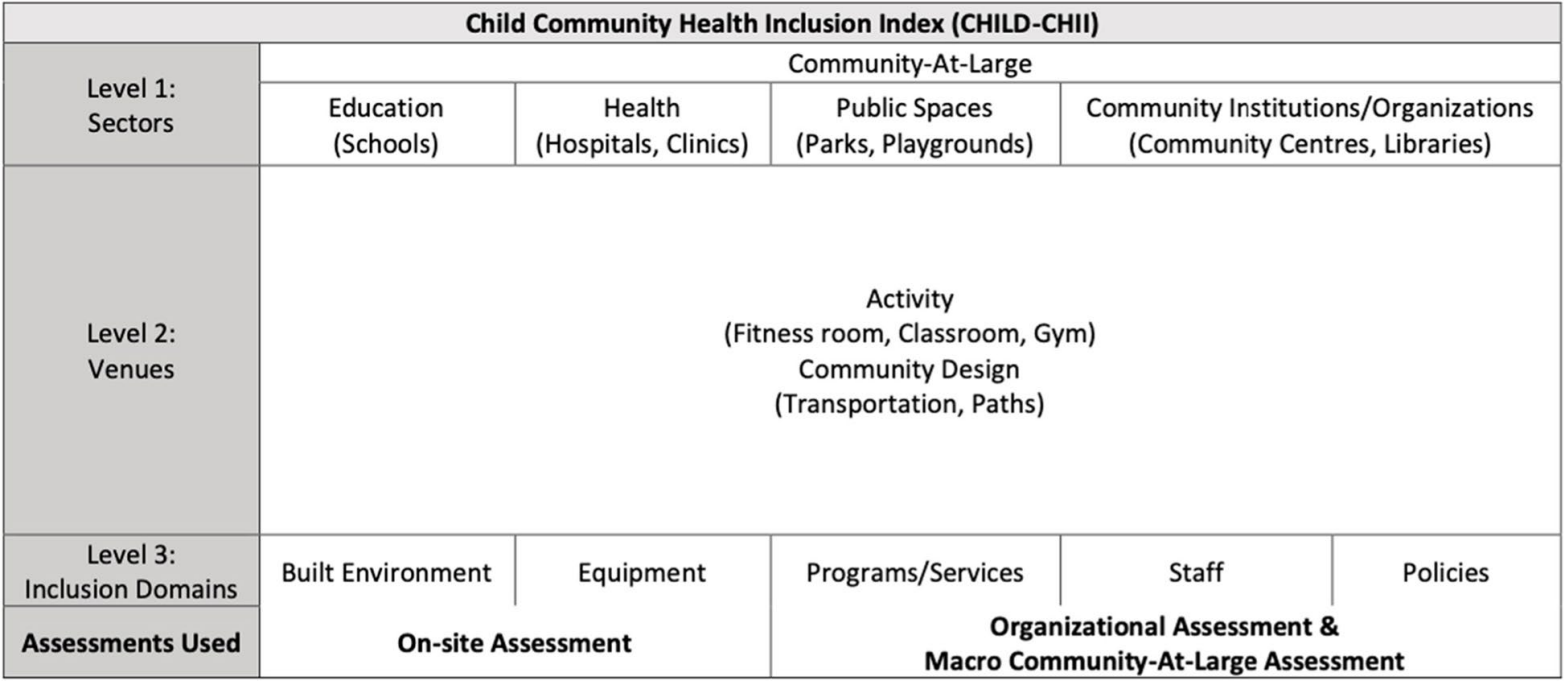
CHILD-CHII Framework.

Participants were recruited through purposeful sampling, from each of the four community sectors: Health, Education, Public Spaces, and, Community Organizations, as described in the previous study of content developed and depicted in the CHILD-CHII framework (Fig. 1) in Quebec, Canada. This included from the ‘Health’ sector- rehabilitation clinicians, program managers and staff providing care and services for children with disabilities; from the ‘Education’ sector- teachers and administration staff working with children with disabilities; for ‘Public Spaces’- staff from municipalities working in inclusion and family policy; for ‘Community Organizations’- program coordinators and staff of organizations that offer activities and services for children with disabilities. Participants were contacted through email with a consent form that included a thorough description of the study and asked to consent to participation. Although an a priori sample size calculation was not made for this feasibility study [7–9], discussions with a pre-established expert panel [5] deemed a sample size of 2–3 participants per sector was adequate to test the feasibility of the tool.

### Measurement tool

The CHILD-CHII is comprised of three assessments that address different inclusion domains of a single facility (Fig. 1). The On-site Assessment consists of 49 items that address the ‘Built Environment’ and ‘Equipment’ inclusion domains of the CHILD-CHII. The Organizational Assessment with 25 items and the Macro Community-At-Large Assessment with 27 items address the inclusion domains of ‘Programs/Services’, ‘Staff’, and ‘Policies’ as related to the facility and the surrounding community.

### Procedures

The three assessments of the CHILD-CHII were provided to the participants in a portable document format that could be printed and completed by hand, and as an online version on the Google Forms platform, in both English and French. The CHILD-CHII manual and the Glossary of Terms were also sent to the participants in English and French. The participants were asked to apply the tool to the facility where they work or are affiliated with and record the total time of application. After the application of the tool, the primary author held semi-structured interviews with each participant by telephone or Zoom to discuss their experience applying the tool during which the participants were asked to rate each feasibility indicator and expand on their ratings. A separate self-reported online demographic questionnaire was sent to each participant after the interview using the Google Forms platform.

### Measures

Feasibility was measured as four indicators addressing the length of the tool, difficulty obtaining the information in order to respond to the items, clarity of the items in the real-life settings, and value of the tool and the information generated for the evaluators and their institutions. These indicators were established a priori based on important aspects of applying the measurement tool that were brought up during the content development and validation phases of the CHILD-CHII [5, 6]. Each feasibility indicator was rated on a 5-point Likert scale (ie. Length- Much too long, …, Much too short; Difficulty- Very easy, …, Very difficult; Clarity- Very clear, …, Unclear; Value- Very valuable, …, Not valuable). Participants had the opportunity to expand on their ratings by adding comments. Participants were asked to expand on their ratings and comments at the follow-up interview and asked probing questions related to each indicator: Length- “Why did you find it long/short?”; Difficulty- “What made it easy/difficult?”; Clarity- “Why did you find it clear/unclear?”; Valuable- “Why do you find the tool valuable/not valuable?”.

### Data analysis

For the ratings of the feasibility indicators, descriptive analyses were performed to characterize the feasibility of the CHILD-CHII measurement tool. Comments and answers to the probing questions provided by the participants were transcribed and then summarized. Suggestions and comments related to the feasibility and refinement of the CHILD-CHII were discussed and the content was analyzed by the first and senior authors.

## Results

A total of 12 participants applied the tool to the facilities in which they work. Table 1 displays the participants’ characteristics.

**Table 1:** Participant characteristics

Characteristic	n	%
Gender		
Female	11	92%
Male	1	8%
Profession/Role		
Clinician	3	25%
Parent	2	17%
Government	2	17%
Community	4	33%
Other	1	8%
Community Sector		
Education	2	17%
Health	3	25%
Community Organization/Institution	5	42%
Public Spaces	2	17%
Experience working with children with disabilities		
Less than 1 year	1	8%
1–3 years	1	8%
4–9 years	3	25%
10–15 years	1	8%
16 + years	2	17%
Not applicable	3	25%
Years working at the current facility		
Less than 1 year	2	17%
1–3 years	1	8%
4–9 years	2	17%
10–15 years	4	33%
16 + years	0	0%
Not applicable	2	17%

### Length

The mean time it took to complete the CHILD-CHII was 1 h and 12 min (Range = 30 min – 3 h). All but one participant indicated that the length of the tool was ‘Long’ (67%) or ‘Much too long’ (25%) (Table 2). The one participant who thought the length was ‘just right’, indicated that they were able to answer the items based on knowledge of the facility and assumptions. They were not able to physically go into the facility to assess due to the SARS-CoV-2 (COVID-19) pandemic. They did mention that if they were to go and retrieve specific information on the items, it may have taken longer.

**Table 2:** Ratings for feasibility indicators

Feasibility Indicator	n	%
Length		
Much too long	3	25%
Long	8	67%
Just right	1	8%
Short	0	0%
Much too short	0	0%
Difficulty		
Very easy	2	17%
Easy	4	33%
Neutral	2	17%
Difficult	3	25%
Very difficult	1	8%
Clarity		
Very clear	4	33%
Clear	4	33%
Neutral	1	8%
Somewhat clear	3	25%
Unclear	0	0%
Value		
Very valuable	4	33%
Valuable	3	25%
Moderately valuable	5	42%
Slightly valuable	0	0%
Not valuable	0	0%

The length was predominantly linked to the scope of the tool with its three assessments, being perceived as too much of a time commitment by the participants. The fact that parts of the tool require information from other sources like coordinators, managers, and potentially community representatives in addition to web searches, made the assessment take a long time to complete, given the need to find the appropriate sources who would have the information. Hence, the items that the participants were able to respond to by themselves did not take too long but the items that required information from other sources took much longer to obtain. Participants stated that highlighting the type of information that may be required and having examples of the people who may have that information, at the beginning of each assessment, would facilitate the process. Furthermore, making it clear that some parts may be skipped if not related nor applicable to the facility, would also reduce the time taken to complete the index.

Participants suggested that the order of the items should be rearranged in a logical sequence to reduce the time. For example, it was mentioned that having items that require the physical evaluation of spaces that are close to one another should be placed subsequently. As well as items or sections that address specific areas of a facility should be grouped and placed sequentially (e.g. items related to the parking lot leading to items addressing the entrance to the facility, then to features of the door).

### Difficulty

The ratings for the difficulty of gathering the required information to complete the tool were quite heterogenous (Table 2). Based on the comments by the participants, the difficulty level was dependent on the evaluator’s familiarity with the concepts brought forth in the tool. Participants who were already working in the field of accessibility for their institution found the items easier to obtain information for completion. Additionally, participants in the coordinator or managerial positions as well as government staff were able to respond to items addressing the ‘Programs/Services’, ‘Staff’, and ‘Policies’ inclusion domains without difficulty. In contrast, clinicians and participants working more directly with children with disabilities found those items more difficult to gather information on and respond. However, participants working directly with children with disabilities found it easier compared to coordinators, managers, and government staff to respond to items related to the ‘Built Environment’ and ‘Equipment’ inclusion domains.

Clinicians and community organization staff reported that it was easier to respond to the On-Site assessment, as they were well aware of their specific institution. Participants generally found the Organizational and Macro Community-At-Large assessments more difficult to complete as they did not have access to the information and simply did not possess the knowledge related to the Programs/Services’, ‘Staff’, and ‘Policies’ inclusion domains; identifying the proper people who have access to the information required to respond to the items in these assessments were reported to be difficult. Subsequently, finding the information to respond to these questions were also reported to be difficult.

Parent participants completed the tool based on their knowledge of the institution and did not apply the tool physically at the site due to the ongoing COVID19 pandemic. Hence, based on this fact, they reported that the completion of the tool was not difficult, but they did foresee its difficulty if and when they would need to apply it on-site.

Both clinician and parent participants mentioned that it was and would be difficult to go further beyond their self and close colleagues to obtain the required information. For clinicians, due to the large patient caseload and paperwork already part of their daily work, it was difficult to take the time to obtain all the required information. For parents, they stated that it would be difficult to reach out to other people and research the information while caring for their child with disabilities and other life tasks.

Having the option to complete the tool on the online platform was mentioned to be useful and participants stated that an online version of the CHILD-CHII should be further refined and used in future versions.

### Clarity

Eight participants (66%) indicated that the items in the tool were ‘Clear’ (33%) or ‘Very clear’ (33%) in what they were asking. Participants reported that access to the glossary was helpful in clarifying some of the terminology (Table 2). One participant who rated the items as ‘Somewhat clear’ stated that they did not know of the glossary initially and referred to the glossary afterward and mentioned that the glossary did make it clearer. The need for the existence of the glossary to be highlighted in the manual and the tool itself was reported.

It was also mentioned that the purpose and objectives of each assessment type (ie. On-Site, Organizational, Macro Community-At-Large) should be clearly stated and defined at the beginning of each section, which will inform and help clarify what the evaluator will be doing and achieving in the particular section.

Participants also found items addressing specific rooms and places of a facility to be unclear in terms of which rooms and places should be considered when responding to the items. For example, for facilities with multiple bathrooms, it was unclear which one should be chosen to answer the items in the ‘Bathroom’ section. They were unsure if they had to respond for all the bathrooms in the facility or a single one. In most cases, the multiple bathrooms had different levels of inclusive features.

### Value

Seven participants (58%) rated the information gathered by the tool to be ‘Valuable’ (25%) or ‘Very valuable’ (33%) (Table 2). These participants reported that the tool is valuable for facilities that are looking to make changes in their accessibility and inclusion and gives a good understanding of the current state of the facility and what is in and around it. Participants valued the items and how they built upon one another while addressing the overarching concept of inclusion. One participant mentioned that it was “thought-provoking” and led to reflections on their “own interventions and practices”. The tool itself brought to light some aspects of the facility that were important for inclusion of children with disabilities, but they were not aware of, which they appreciated. A participant from the government sector mentioned that the tool could be a common platform that connects the different departments within the municipal government- the building department, engineering department, and inclusion department- the assessment would be “helpful to bring everyone together”. Participants also reported that the CHILD-CHII shed light on areas of the facility that they did not previously think about with regards to inclusion of children with disabilities (e.g. Public transportation routes and accessible signage for getting around inside the facility).

Five participants (42%) rated the information to be ‘Moderately valuable’ (Table 2). These participants were involved in the accessibility/inclusion sphere of their corresponding institutions and stated that they were already aware of most of the things outlined by the tool- “being in the field, [participant] already know the thing that need to be changed”, “tool was not necessarily needed to know what to change”. For community organizations that are solely focused on accessibility and inclusion, “accessibility and inclusion are already considered” and the facility was built with accessibility and inclusion in mind. However, these participants did state that the tool and the information gathered by the tool would be valuable and helpful to facilities that are not involved in the field and would require support in establishing accessibility and inclusion.

One interesting theme brought forth by the participants was in regard to the possibility of change and their capacity to make a change. They found that some aspects of the assessment, especially the inclusion domains related to ‘Programs’, ‘Staff’, and ‘Policies’ is “too removed from what [they] can do” and “directly impact”. This theme was more prominent among clinicians and community organization staff who worked more closely on the ground. Some of the aspects of the tool are “beyond the possibility of the institution” or the individual and could be discouraging for the evaluator as some evaluators “do not have the power to make a change”. If the evaluator does not see that they are able to make a change within a certain domain, they may not find “value or worth for the evaluator”; some “would not be able to do anything with the information”. However, participants did find that the results of the tool “can be brought to a higher manager to target and address the gaps that were found” and saw that it can be used to advocate for change and as resource supports, perhaps with several other facilities within a municipality or region.

Clinician and parent participants stated that having more opportunities to expand on certain responses to items, in the form of a comment section, would make the tool more valuable. Parents also mentioned that having access to the scores of the facilities in their local community would be valuable to access for their consideration.

Parents highlighted the value of being more specific in addressing the aspects of the facility while the other participant groups found that being too lengthy for their scope of practice could be of less value. Most participants mentioned that the perceived value of the tool and the information gathered would influence the time and effort put into completing the tool.

## Discussion

This study aimed to estimate the feasibility of the CHILD-CHII measurement tool and its application on community facilities. The length of the CHILD-CHII was found to be long or much too long by the participants. The difficulty of completing the tool were more specific to the evaluator, hence, the comments offered pertinent and valuable indication of the difficulty. In terms of clarity of the items, most participants found the tool to be clear or very clear. None of the participants rated the tool as not valuable and a majority rated the index to be valuable or very valuable.

The participant’s knowledge, familiarity, and access to the information required to complete the tool were major factors in influencing the feasibility of using and applying the tool, especially with regards to the perception of length and difficulty of applying the CHILD-CHII. This is consistent with evidence indicating that the knowledge, understanding, and familiarity of outcome measures increase the likelihood of their use in practice amongst healthcare professionals [10]. Participant knowledge and experience with inclusion and accessibility led to easier application of the tool. The familiarity with the concepts and topics addressed by the items in each of the assessments made it faster and easier for the participants to respond. As such, having multiple evaluators complete different sections of the tool based on their knowledge and role would shorten the time to complete the tool with adequate information at hand.

The time it takes to complete a measure was found to be a major barrier in its use by both healthcare professionals and community organizations [11, 12]; in both developing and developed countries [13]. With this considered, the reported perceived length of the tool found in this study suggests a reduced feasibility in applying the tool. However, the participants stated that the perceived value of the tool influences the worth of time and effort required in completing the tool. This is consistent with literature that found that the perceived value and relevance facilitates the use of any outcome measure [14]. The importance of each of the items included in the tool was rated and considered in the content validation of the CHILD-CHII which involved participants from multiple stakeholder groups including clinicians, families of children with disabilities, government and community workers [6]. This was further affirmed as all of the participants in this study indicated that the CHILD-CHII was moderately to very valuable. This may indicate a higher likelihood to utilizing the tool despite the time required to complete the tool. Furthermore, the efforts to clarify each item included in the tool, that was also undertaken with the input of multiple stakeholders during the content validation of the CHILD-CHII, ensured the clarity of the CHILD-CHII [6].

Having an online version of the CHILD-CHII was found to be beneficial and easier to apply for some participants. The option of completing the CHILD-CHII on a web-based platform can make it more feasible to access the glossary (using hyperlinks) and use and apply for evaluators [15]. In another study, participants also preferred electronic versions as opposed to paper-based for patient-reported outcome measures [16].

The context in which the evaluator is situated could be both a facilitator and barrier to the use of this outcome measure [10, 17]. Organizational factors like the priority and focus on inclusion set by the organization can augment the use of the measure. Hence, having the organizational support in prioritizing inclusion and allocating time for assessing inclusion of the facility could support the feasibility of the CHILD-CHII use [10, 11]. This highlights the importance of having an institutional and cultural shift in the prioritization of inclusion and accessibility as an integral part of clinical and policy interventions. Stakeholders value the use of measurement tools when the tools are useful for their decision-making process or in strategic planning [15]. This was evident in this feasibility study as participants in decision-making positions (i.e. managers, coordinators, government workers) found that the results of the CHILD-CHII would be beneficial for their facilities and institutions in implementing further actions to improve their inclusion; and as participants mentioned, shed light on areas and aspects of the facility that they did not consider with regards to inclusivity.

This study demonstrated the potential for the CHILD-CHII in providing awareness about important gaps and potential actionable solutions for the inclusion of children with disabilities, how it can be achieved, and where improvements need to be made. The specificity of the items can provide specific suggestions that could be included in policies and program planning at the level of the individual child in health and education, and at the macro level of building communities and improving universal accessibility. The measurement of these aspects can generate indicators put forth by all major international agendas and organizations such as the World Health Organization Urban Health Agenda and the International Classification of Functioning, Disability, and Health [18, 19]; the UN CRPD, CRC, and Sustainable Development Goals [1, 2, 20]; and the public health Community Well Being framework [21]. Awareness about how to integrate the CHILD-CHII as a benchmark into these agendas would further facilitate the use of the tool. With modifications based on the comments and suggestions made by the participants in this study, the CHILD-CHII measurement tool will be ready for a larger psychometric study to establish the reliability and validity of the tool.

### Limitations

One limitation of this study was the small sample size that was recruited through convenience sampling of participants in Quebec, Canada. Although the results may not be generalizable, the sample was adequate to provide a good indication of the feasibility of applying the CHILD-CHII while providing important comments that will be implemented in refining the measurement tool, prior to further psychometric testing. The skewed female representation of the small sample may also have implications in feasibility that were not addressed in this study. The second limitation is the lack of participants from the Education sector. Although we had clinicians working in the school setting, teachers could not be recruited after much effort. This gap in participation from the education sector may indicate the challenge of coordinating health and education sectors when providing services for children with disabilities, and may also highlight the variability with respect to how different sectors prioritize accessibility and inclusion.

## Conclusion

This study on the feasibility of applying the CHILD-CHII provided insights on its use in community settings. Ratings of clarity of the items in the tool were confirmed to be well-defined. Although the tool was regarded as long, the information gathered and provided by the tool was seen to be valuable for community facilities that provide services and programs for children with disabilities. The knowledge, familiarity, and access to the information of the evaluators should be considered and the scope of each assessment should be clearly provided to make it easier for evluators to use the CHILD-CHII. The CHILD-CHII can be a viable and valuable tool to inform strategic planning, involving multiple stakeholder groups including clinicians, parents, institutions, community organizations, and policymakers in addressing and improving the inclusion of the children with disabilities in the community to foster community participation and healthy living.

## Supplementary Information

**Additional file 1.** Child Community Health Inclusion Index.

## Data Availability

The datasets used and/or analysed during the current study available from the corresponding author on reasonable request.
