# Supplementary material for: Application of the child community health inclusion index for measuring health inclusion of children with disabilities in the community: a feasibility study
Source: BMC Pediatr. 2023 Feb 20;23:86. doi: 10.1186/s12887-023-03884-8 (PMC9939852; doi:10.1186/s12887-023-03884-8)
Supplement: Supplementary file 1 — Additional file 1. Child Community Health Inclusion Index. [file 12887_2023_3884_MOESM1_ESM.pdf]

Child Community Health Inclusion Index

Table of Contents

Child Community Health Inclusion Index 2

On-site Assessment 2
Before Heading to the Site 2
Transit 3
Getting Around the Site 4
Inside the Site 7
Bathroom 10
Information 11
Emergency 13
Guide Dogs 13
Locker Rooms 13
Exercise Equipment/Space 14
Pools 15
Playgrounds 16
Water Fountain/Splash Pad 17
Multi-use Trail 18
Waiting Room 18
Exam Room 19
Final Overall Questions 19

Organizational Assessment 20
Information about the Site 20
Activity 21
Materials 23
Staff 23
Policymaking 25
Wayfinding 25
Schools 26
Healthcare Sites 26
Readiness for Change 27
Audit 29

Macro Community-At-Large Assessment 30
Information about the Site 30
Transportation 30
Transportation Staff Training 32
Community Design 33
Awareness Initiatives 34
Healthcare Access 34
General Programs/Services 35
Leisure 35
Volunteer/Work 35
Education 36
Social 36
Technology 37
Web Mapping 38
Accessibility Policies 38

Child Community Health Inclusion Index

On-site Assessment

Thank you for taking the time to fill out this assessment about the accessibility and inclusion of a facility in your community for children with disabilities.

The purpose of the Community Health Inclusion Index (CHII) is to collect information about healthy living resources in a community and the degree to which they are inclusive. The assessment will be extremely useful in helping stakeholders to plan and develop strategies that will impact the health and well-being of all members of your community, including children with disabilities.

Please note that the CHILD-CHII is not a compliance checklist, but is intended to be a tool to measure a broad level of health inclusion in communities. It may take 30-60 minutes, depending on the site.

The CHILD-CHII On-Site Assessment is designed in sections. Please make sure to review all the sections of the survey and answer all that apply to the site being assessed. There are instructions and pictures to help conduct the assessment. In addition, raters should review the CHILD-CHII Manual for additional instructions on rating sites, measurement, and a glossary of terms.

Before Heading to the Site

Fill out the following information before heading to the site.

Rater Name: ____

Facility Name: ____

Street Address:
____

____

City: ____

Province: ____

Zip Code: ____

Choose the Sector of the community that most applies to the site being assessed.

- O Education
- O Health
- O Public Spaces
- O Community Institutions/Organizations

Transit

1. Is there at least one public transit stop near the site entrance?

- O Yes
- O No

If yes to the previous question, how close?

- O Less than 50 m
- O 50 - 100 m
- O More than 100 m

2. Which of the following components are available on public transportation vehicles that have a stop near the site? Check all that apply.

- ☐ Level boarding from ramp or lowered vehicle
- ☐ Auditory announcements
- ☐ Visual display of stops
- ☐ Other: ____

3. Which of the following elements, if any, are observed at the public transit stop? Check all that apply.

   - ☐ Transit shelter, bench or other seating
   - ☐ Signage with TTY number (Telecommunication Device for the Deaf)
   - ☐ Enough space to maneuver using a mobility device
   - ☐ Stable and firm landing pad surface
   - ☐ Light posts or other lighting infrastructure at or next to the stop
   - ☐ Other: ____

Getting Around the Site

4. Assess the Path from the transit stop to the site if available, otherwise, complete for the path from the parking area. Check all that apply.

   - ☐ At least 5 feet wide
   - ☐ Free of obstacles or hazards that are difficult to traverse
   - ☐ Surface smooth and firm
   - ☐ Other: ____

5. Indicate if the following characteristics that make getting around (outside) the site more accessible are present. Check all that apply.

   - ☐ Some form of physical boundary around common areas that lessens the likelihood of children wandering into unsupervised areas
   - ☐ Wide aisles
   - ☐ Obstacles and edges are detectable for someone using a cane
   - ☐ Routes are free of obstacles
   - ☐ Slip-resistant materials

☐ Circle or a T-shaped space for a person/child using a wheelchair to reverse direction or to turn their wheelchair completely

☐ Contrasting colours

☐ Even terrain

☐ Other: ____

6. Are there any driveways, street crossings, or changes in levels on the path to the site where a curb cut (pavement graded down to meet lower, adjoining street) is needed but does not currently exist?

   ○ Yes

   ○ No

7. For the curb cuts anywhere on a path, indicate whether the following characteristics are observed. Check all that apply.

   ☐ Gradual slope

   ☐ Free of barriers or hazards that obstruct it

   ☐ Free of breaks in the surface

   ☐ Detectable warning in good working condition

   ☐ Tactile warning panels

   ☐ Other: ____

8. Indicate whether the following features are present in the pedestrian crossings near the site. Check all that apply.

   ☐ Crosswalk is well marked with stripes/paint/bricks

   ☐ Free of obstacles or hazards that are difficult to traverse

   ☐ Curb cuts at each end of the crossing

- ☐ Tactile paving guides
- ☐ Other: ____

9. Do the intersections around the site have a traffic signal?

- ○ None of them
- ○ Less than 50% of them
- ○ 50% of intersections
- ○ More than 50% of them
- ○ All of them

10. Indicate whether the following elements are present on the traffic signals near the site. Check all that apply.

- ☐ Auditory crossing signal
- ☐ Visual crossing signal
- ☐ Other: ____

11. Is a parking lot available at the site?

- ○ Yes
- ○ No

12. Are any of the following observed in the parking lots? Check all that apply.

- ☐ Accessible spaces designated with International Symbol of Accessibility on an upright sign
- ☐ Access aisles adjacent to accessible parking spaces
- ☐ Designated van accessible parking spaces

☐ Other: ____

13. Is the pathway towards the site clear of ice and snow?

○ Yes

○ No

○ Not applicable

Inside the Site

14. Are the following accessible features present at the main entrance? Check all that apply.

☐ Ramp

☐ Lift

☐ Non-slip surface

☐ Tactile indication for steps and ledges

☐ Stairs with continuous rails on one or both sides

☐ Power assist or automatic door

☐ Other: ____

15. If no adaptations at the main entrance, does an alternate accessible entrance exist?

○ Yes

○ No

○ Not applicable

If yes, check all that apply.

☐ Ramp

☐ Lift

☐ Non-slip surface

☐ Tactile indication for steps and ledges

☐ Stairs with continuous rails on one or both sides

☐ Power assist or automatic door

☐ Other: ____

16. Can the alternate entrance be used without additional assistance?

    ○ Yes

    ○ No

    ○ Not applicable

17. Are the accessible drop-off areas/parking spaces closest to the accessible entrance?

    ○ Yes

    ○ No

    ○ Not applicable

18. Do all inaccessible entrances have signs indicating the location of the nearest accessible entrance?

    ○ Yes

    ○ No

    ○ Not applicable

19. On each floor of the site, is there an accessible route to all the essential public areas (Eg. Bathroom, Emergency exit)?

   - ○ Yes
   - ○ No

20. Do the doors at the site have the following accessible features? Check all that apply.

   - ☐ Automatic operation
   - ☐ Sliding doors
   - ☐ Wide enough for a wheelchair
   - ☐ Flush threshold (level doorway)
   - ☐ Other: ____

21. Which of the following features are found in rooms and shared spaces (accessed by the child) at the site. Check all that apply.

   - ☐ Facilitated communication with people who use sign language (e.g. people facing each other)
   - ☐ Appropriate acoustics
   - ☐ Noise reduction measures in place
   - ☐ Adequate lighting
   - ☐ Other: ____

22. Are any of the following present for navigating around the site? Check all that apply.

   - ☐ Elevator
   - ☐ Ramp
   - ☐ Lift

☐ Tactile indicators

☐ Other: ____

23. Does the signage for navigating around the site have any of these features? Check all that apply.

☐ High-contrast lettering

☐ Large print

☐ Pictograms

☐ Braille

☐ Other: ____

Bathroom

24. Is there a bathroom that is fully accessible?

○ Yes

○ No

25. Are the following accessible features found with bathroom entrances at the site? Check all that apply.

☐ Automatic operation

☐ Open corridor entrance

☐ Wide enough for a wheelchair

☐ Flush threshold (level doorway)

☐ Other: ____

26. Are the following accessible features used to identify bathrooms at the site? Check all that apply.

- ☐ Pictograms/Symbols
- ☐ Raised characters
- ☐ Braille
- ☐ Low enough for children to see
- ☐ Other: ____

27. Are the following accessible features found in bathrooms at the site? Check all that apply.

- ☐ Adequate space for up to two caregivers
- ☐ Adult-sized changing station
- ☐ Adapted toilet with grab-bars
- ☐ Tilted mirror
- ☐ Low sink
- ☐ Low hand dryer
- ☐ Low soap dispenser
- ☐ Other: ____

Information

28. Are information materials (Eg. Pamphlets, Flyers) offered in any of the following formats? Check all that apply.

- ☐ Electronic version in plain text
- ☐ Large print
- ☐ Pictograms

☐ Audio

☐ Braille

☐ Not applicable

☐ Other: ____

29. Do promotional materials for programs indicate the program is inclusive of children with disabilities (through images of individuals with disabilities participating or descriptions of the programs?)

○ Yes

○ No

○ Not applicable

If yes, please describe.

____

____

____

30. Which of the following accessible features are found at the front/information desk? Check all that apply.

☐ Low enough for children to access

☐ Tactile cues

☐ Large color contrasted signage

☐ Other: ____

Emergency

31. Which of the following emergency features are found at the site? Check all that apply.

- ☐ Emergency call/help points
- ☐ Emergency alerts with lights
- ☐ Emergency alerts with sounds
- ☐ Other: ____

Guide Dogs

32. Is there a designated space for guide dogs at the site?

- ○ Yes
- ○ No

Locker Rooms

33. Which of the following inclusive features do the locker rooms have? Check all that apply.

- ☐ Not applicable
- ☐ Wide entrance
- ☐ Locker door handles can be reached by an individual seated in a mobility device
- ☐ Paths in the locker room are free of obstacles
- ☐ Clear space in front of lockers
- ☐ Other: ____

34. Is there an accessible family change room at the site?

- O Yes
- O No

Exercise Equipment/Space

35. Is adaptive equipment available for children with disabilities to participate in given activities?

- O Not applicable
- O Yes
- O No

If yes, please list the equipment.

____

____

____

36. Are child-friendly instructions for the use of the equipment readily available and accessible?

- O Not applicable
- O Yes
- O No

If yes, please describe.

____

____

____

37. Which of the following features do the aisles/paths in the activity area have?

- ☐ Wide enough for mobility devices
- ☐ Free of obstacles or hazards that are difficult to traverse
- ☐ Tactile cues
- ☐ Not applicable
- ☐ Other: ____

38. What opportunities do children with disabilities have to engage in exercise/fitness (eg. adapted exercise activities)? Please describe. (Skip if not applicable)

____

____

____

Pools

39. Which of the following features are present at the pool? Check all that apply.

- ☐ Not applicable
- ☐ Zero-depth entrance
- ☐ Ramp or lift to enter
- ☐ Floatation devices
- ☐ Slip-resistant flooring around the pool
- ☐ Heated/therapeutic section of the pool
- ☐ Contrasting colours
- ☐ Tactile cues
- ☐ Large pictograms

- ☐ Large signs/indicators
- ☐ Other: ____

Playgrounds

40. Which of the following features does the playground have? Check all that apply.

- ☐ Not applicable
- ☐ Ground material that can be traversed using a mobility device
- ☐ Large signage/pictograms at child-friendly height
- ☐ Tactile map
- ☐ Other: ____

41. Which of the following features does the playground equipment have? Check all that apply.

- ☐ Not applicable
- ☐ Varying heights for use by children
- ☐ Knee clearance providing wheelchair access
- ☐ Accessible reach ranges
- ☐ Sensory elements
- ☐ Other: ____

If any sensory elements, please describe them.

____

____

____

42. Which of the following features are found around the playground? Check all that apply.

- ☐ Bench
- ☐ Picnic table
- ☐ Shelter/Shade
- ☐ Drinking fountain
- ☐ Accessible bathroom
- ☐ Other: ____

Water Fountain/Splash Pad

43. Which of the following features are present at the water fountain/splash pad? Check all that apply.

- ☐ Not applicable
- ☐ At least one fountain with clear floor space
- ☐ Tactile cues on floor
- ☐ Varying fountain heights, some low enough for wheelchair access
- ☐ Control mounted on child-friendly height
- ☐ Benches or rest area
- ☐ Accessible bathroom nearby
- ☐ Other: ____

Multi-use Trail

44. Which of the following features does the multi-use trail have? Check all that apply.

- ☐ Not applicable
- ☐ Benches or rest areas
- ☐ Firm, smooth surface
- ☐ Wide enough for a wheelchair
- ☐ Free of obstacles or hazards that may be difficult to traverse
- ☐ Navigational aids, such as pictograms/signage
- ☐ Tactile cues
- ☐ Other: ____

Waiting Room

45. Is there a waiting room available at the site?

- ○ Yes
- ○ No
- ○ Not applicable

46. Which of the following features are present in the waiting room? Check all that apply.

- ☐ Not applicable
- ☐ Wide enough for a wheelchair
- ☐ Free of obstacles or hazards
- ☐ Interactive screens
- ☐ Toys

- ☐ Other: ____

Exam Room

47. Which of the following features does the exam/diagnostic room have? Check all that apply.

- ☐ Not applicable
- ☐ Transfer support available for moving to exam table, such as transfer board or lift
- ☐ Adjustable exam table
- ☐ Sufficient space provided for maneuvering inside the room in a mobility device
- ☐ Weighing scale that has railings for stability
- ☐ Wheelchair-accessible scale that can accommodate children and their wheelchair
- ☐ Other: ____

Final Overall Questions

48. Are there any other aspects of the site that are supportive for children with a disability to participate in the activity?

____

____

____

49. Are there any other aspects of the site that are a barrier (hinder or prevent) for children with a disability to participate in the activity?

____

____

____

Organizational Assessment

Thank you for taking the time to fill out this survey about the accessibility and inclusion of the organization in regards to promoting the inclusion of children with disabilities. The purpose of the Child Community Health Inclusion Index (CHILD-CHII) is to collect information about healthy living resources in the community and the degree to which they are inclusive. The assessment will be extremely useful in helping stakeholders to plan and develop strategies that will impact the health and wellbeing of all members of the community, including children with disabilities.

Important Note:
It may be helpful to complete the survey with other people in the organization who may be able to address different areas. The CHILD-CHII Organizational Assessment will take around 15-30 minutes to complete depending on what is available at the site.

Information about the Site

Name of the Organization/Site: ____

Address:
____

____

City: ____

Province: ____

Zip Code: ____

Choose the Sector of the community that most applies to the site being assessed.

- ○ Education
- ○ Health
- ○ Public Spaces
- ○ Community Institutions/Organizations

Please describe the role of the person with whom this assessment is being completed, if applicable.

- ☐ Supervisor/Manager/Owner
- ☐ HR Coordinator
- ☐ Communication Liaison
- ☐ Teacher
- ☐ Other: ____

Activity

1. Is at least one activity program/class available on-site for children with disabilities?

   - ○ Yes
   - ○ No
   - ○ Not applicable

2. Are activities/programs held in an accessible location?

   - ○ Yes
   - ○ No
   - ○ Not applicable

If yes, please specify which activities/program.

____

____

____

3. Is the activity/program designed so that children with disabilities and without disabilities participate equally, such as adapting movements and rules?

   - Yes
   - No
   - Not applicable

4. Are accommodations provided so children with disabilities can participate, such as allowing an aide or caregiver to attend?

   - Yes
   - No
   - Not applicable

5. Are there any other aspects related to activity at the site that are either supportive or may be a barrier to persons/children with disabilities?

   - Yes
   - No
   - Not applicable

If yes, please specify.

____

____

____

Materials

6. Are any Instructional/Educational materials regarding activities/program(s) available to children and families?

   ○ Yes

   ○ No

   ○ Not applicable

7. Which of the following alternative formats are readily available for the Instructional/Educational materials? Check all that apply.

   ☐ Not applicable

   ☐ Braille

   ☐ Electronic version

   ☐ Large print

   ☐ Pictograms

   ☐ Audio

   ☐ Video with captions

   ☐ Other: ____

Staff

8. Are staff provided any type of disability awareness training either on-site or through outside education?

   ○ Yes

   ○ No

9. Which of the following components are covered in disability awareness training? Check all that apply.

- ☐ Not applicable
- ☐ Providing services to children with different types of disabilities and/or their families
- ☐ Adapting the environment for children with disabilities
- ☐ Communicating with children/parents of children with different types of disabilities
- ☐ Using person-first terminology
- ☐ Other: ____

10. Which of the following policies apply to the disability awareness training? Check all that apply.

- ☐ Not applicable
- ☐ Disability awareness training is part of human resource policies
- ☐ Staff at all levels receive disability awareness training
- ☐ Persons with disabilities are involved in providing the training
- ☐ Other: ____

11. Which of the following information is included in the training materials that are available to the staff? Check all that apply.

- ☐ Not applicable
- ☐ Facing different groups of children and recommendations on how to facilitate participation for these groups
- ☐ Emergency situations that may arise
- ☐ Comprehensive overview of barrier-free participation
- ☐ Definitions and/or descriptions of different types of disabilities

- ☐ Basic sign language
- ☐ Strategies for conflict resolution with children and/or family members
- ☐ Other: ____

Policymaking

12. When organizing committee groups for overall policymaking at the facility, are children with disabilities and/or parents of children with disabilities included?

    - ○ Yes
    - ○ No

If yes, please describe their involvement.

____

____

____

Wayfinding

13. Which services are available to help children with disabilities navigate around the facility? Check all that apply.

    - ☐ Personalized mapping photos/schemes with labels
    - ☐ Accompaniment by a staff
    - ☐ Other: ____

Schools

14. Are there accommodation programs available for children with disabilities to get to school?

- ○ Not applicable
- ○ Yes
- ○ No

If yes, please describe the program.

____

____

____

15. Which of the following policies on inclusion are adopted by the school (or school district)? Check all that apply.

- ☐ Not applicable
- ☐ Physical activity goals are included in students' Individual Education Programs (IEPs)
- ☐ Adapted sports program are available in the school
- ☐ Students of all abilities participate in PE class together
- ☐ Other: ____

Healthcare Sites

16. Do healthcare providers ask children with disabilities about their level of physical activity?

- ○ Not applicable
- ○ Yes
- ○ No

17. Are healthcare providers able to weigh a child using a mobility device who is unable to stand using a roll-on or lift scale?

- ○ Not applicable
- ○ Yes
- ○ No

Readiness for Change

18. How aware is the organization about the inclusion of children with disabilities in health promotion? (1 being not at all aware; 5 being very aware)

- ○ 1
- ○ 2
- ○ 3
- ○ 4
- ○ 5

19. How much of a concern is inclusion in health promotion in your organization? (1 being no concern at all; 5 being very great concern)

- ○ 1
- ○ 2
- ○ 3
- ○ 4
- ○ 5

20. Would the leadership support additional efforts toward inclusion in health promotion?

- ○ Yes
- ○ No

Please explain your answer.

____

____

____

21. Is the organization currently planning for any additional efforts/services towards inclusion in health promotion for persons/children with disabilities?

- O Yes
- O No

If yes, please explain your answer.

____

____

____

22. Have any plans been adopted for making the building/site more accessible?

- O Yes
- O No

If yes, please explain your answer.

____

____

____

23. What are the primary obstacles to efforts addressing inclusion of children with disabilities in health promotion in the organization?

____

____

____

Audit

24. Is there an accessibility auditing in place? (an assessment of a building, best-practice standards to benchmark its accessibility)

    - Yes
    - No

25. Is there a mechanism in place to make the changes recommended by the audit?

    - Yes
    - No

If yes, please explain.

____

____

____

Macro Community-At-Large Assessment

The questions in the CHII Macro-Community Assessment are related to transportation services and policies, community design policies and programs, and community wellness initiatives that are found around the facility. Raters answer these questions by conducting online research as well as interviewing local transit agency representatives and community planners. For additional instructions, please see the CHILD-CHII Manual.

Note: it may be helpful to fill this out on paper first while gathering information from various sources and then fill out the answers once you are done compiling.

Information about the Site

Rater Name: ____

Facility Name: ____

Address: ____

____

City: ____

Province: ____

Zip Code: ____

Transportation

1. Is at least one form of fixed route, Public Transportation available in the community, such as a public bus, train, and/or subway?

   - O Yes
   - O No

2. Is there a program in the community that provides travel training for children with disabilities in using public transportation?

   - O Yes

- ○ No
- ○ Not applicable

3. Are there subsidies that are available for public transit for the following groups of people (children with disabilities, low income)?

- ○ Yes
- ○ No

If yes, please describe.

____

____

____

4. Is information on the accessibility of the transit system and stops posted on the transportation agency's website?

- ○ Yes
- ○ No

5. Which of the following accessibility features are provided regarding the information on transportation? Check all that apply.

- ☐ Not applicable
- ☐ Plain text documents
- ☐ Large print
- ☐ Pictograms
- ☐ Tactile map of transportation system
- ☐ Audio

- ☐ Braille
- ☐ Other: ____

6. Which of the following other types of transportation services for children with disabilities are available in the community? Check all that apply.

- ☐ Para-transit (door-to-door)
- ☐ Volunteer transportation service
- ☐ Wheelchair accessible taxis
- ☐ Other: ____

7. Are there support systems in place to help children with disabilities at transportation hubs in the community?

- O Yes
- O No

If yes, please describe.

____

____

____

Transportation Staff Training

8. Does the public transportation staff receive disability awareness training?

- O Yes
- O No
- O Not applicable

9. Which of the following are a part of the staff’s disability awareness training? Check all that apply.

   ☐ Communicating with people with different types of disabilities

   ☐ Using person-first terminology

   ☐ Other: ____

10. Which of the following policies apply to the transportation staff’s disability awareness training? Check all that apply.

   ☐ Disability awareness training is part of human resource policies

   ☐ Staff at all levels receive disability awareness training

   ☐ Persons/children/parents of children with disabilities are involved in providing training

   ☐ Other: ____

Community Design

11. Which of the following inclusive policies or regulations exist in the community? Check all that apply.

   ☐ Development of biking and walking/rolling infrastructure

   ☐ Transit-oriented development of the community

   ☐ Installment of wayfinding signage in the community, such as for biking or walking routes

   ☐ Wayfinding signage inclusive of children with disabilities by having large print, pictograms and Braille

   ☐ Program or service to maintain sidewalks, such as for clearing snow or removing water near curb cuts

   ☐ Funding available in the community to improve accessibility at business locations and community facilities

   ☐ Other: ____

Awareness Initiatives

12. Does a program generating dialogue between children/families of children with disabilities and service providers/policy makers in the community exist?

    - Yes
    - No

13. Does a program generating dialogue between children/families of children with disabilities and service providers/policy makers in the community exist?

    - Yes
    - No

Healthcare Access

14. Are opportunities to access healthcare for a physical health condition readily available in the community?

    - Yes
    - No

15. Are opportunities to access healthcare for a mental health condition readily available in the community?

    - Yes
    - No

General Programs/Services

16. Are there any community groups, clubs, or organizations for children with disabilities in the community?

    - ○ Yes
    - ○ No

Leisure

17. Do leisure opportunities exist for children with disabilities in the community?

    - ○ Yes
    - ○ No

If yes, please describe.

____

____

____

Volunteer/Work

18. Do volunteer/work opportunities exist in the community for children with disabilities?

    - ○ Yes
    - ○ No

If yes, please describe.

____

____

____

Education

19. Are any of the following in the area accessible for children with multiple disabilities? Check all that apply.

- ☐ School
- ☐ Workshops
- ☐ Educational sessions
- ☐ Tutoring
- ☐ Daycare centre
- ☐ Other: ____

Social

20. Are there opportunities in the community for families to participate together in social activities?

- ○ Yes
- ○ No

If yes, please describe.

____

____

____

21. Are there opportunities in the community for children with disabilities to meet other people in the community?

- ○ Yes
- ○ No

If yes, please describe.

____

____

____

22. How would you describe the community members' attitudes towards children with disabilities within the community at large?

____

____

____

Technology

23. Are there opportunities in the community for children with disabilities to access computer technology and technology services?

   - ○ Yes
   - ○ No

If yes, please describe.

____

____

____

Web Mapping

24. Are any mobile Apps such as apps listing activities, accessible buildings, accessible routes, or adapted transit available to the community?

- ○ Yes
- ○ No

If yes, please describe.

____

____

____

Accessibility Policies

25. Are there any accessibility policies and/or initiatives in the community?

- ○ Yes
- ○ No

If yes, please describe.

____

____

____

26. Are there any accessibility or inclusion awards/recognitions or certifications/seals available to the larger community? (e.g. to the municipality)

- ○ Yes
- ○ No
- ○ Not applicable

27. Accessibility Policies Are the following things integrated in overall policymaking in the community? Check all that apply.

- ☐ Organising a round table with policymakers and children with disabilities and/or parents of children with disabilities
- ☐ Presence of Disability Advisory Group, who represent children with a broad range of impairments
- ☐ Other: ____
